# Could *Histoplasma capsulatum* Be Related to Healthcare-Associated Infections?

**DOI:** 10.1155/2015/982429

**Published:** 2015-05-27

**Authors:** Laura Elena Carreto-Binaghi, Lisandra Serra Damasceno, Nayla de Souza Pitangui, Ana Marisa Fusco-Almeida, Maria José Soares Mendes-Giannini, Rosely Maria Zancopé-Oliveira, Maria Lucia Taylor

**Affiliations:** ^1^Departamento de Microbiología-Parasitología, Facultad de Medicina, Universidad Nacional Autónoma de México (UNAM), Circuito Interior, Ciudad Universitaria, Avenida Universidad 3000, 04510 México, DF, Mexico; ^2^Instituto Nacional de Infectologia Evandro Chagas, Fundação Oswaldo Cruz (FIOCRUZ), Avenida Brasil 4365, Manguinhos, 21040-360 Rio de Janeiro, RJ, Brazil; ^3^Departamento de Análises Clínicas, Faculdade de Ciências Farmacêuticas, Universidade Estadual Paulista (UNESP), Rodovia Araraquara-Jaú Km 1, 14801-902 Araraquara, SP, Brazil

## Abstract

Healthcare-associated infections (HAI) are described in diverse settings. The main etiologic agents of HAI are bacteria (85%) and fungi (13%). Some factors increase the risk for HAI, particularly the use of medical devices; patients with severe cuts, wounds, and burns; stays in the intensive care unit, surgery, and hospital reconstruction works. Several fungal HAI are caused by *Candida* spp., usually from an endogenous source; however, cross-transmission via the hands of healthcare workers or contaminated devices can occur. Although other medically important fungi, such as *Blastomyces dermatitidis*, *Paracoccidioides brasiliensis*, and *Histoplasma capsulatum*, have never been considered nosocomial pathogens, there are some factors that point out the pros and cons for this possibility. Among these fungi, *H. capsulatum* infection has been linked to different medical devices and surgery implants. The filamentous form of *H. capsulatum* may be present in hospital settings, as this fungus adapts to different types of climates and has great dispersion ability. Although conventional pathogen identification techniques have never identified *H. capsulatum* in the hospital environment, molecular biology procedures could be useful in this setting. More research on *H. capsulatum* as a HAI etiologic agent is needed, since it causes a severe and often fatal disease in immunocompromised patients.

## 1. Introduction

The term healthcare-associated infection (HAI) refers to infections associated with healthcare delivery in any setting (e.g., hospitals, long-term care facilities, ambulatory settings, and home care). This term reflects the inability to determine with certainty where the pathogen is acquired since patients may be colonized or exposed to potential pathogens outside the healthcare setting, before receiving healthcare or during healthcare delivery [1, 2].

In recent years, there has been an overall increase in HAI, which is likely a consequence of the advances in medical and surgical procedures related to specific therapies, in addition to the large number of immunocompromised patients who are hospitalized [3]. It is estimated that every day one out of 25 hospital patients has, at least, one HAI. In 2011, there were 722,000 HAI in the United States' hospitals and about 75,000 hospital patients with HAI died during their hospitalization. More than half of all HAI occurred outside the intensive care unit [4].

HAI commonly occur by direct transmission from individual to individual or through fomites manipulated by healthcare workers, as well as through surfaces and devices contaminated by biofilms (surgical instruments, catheters, mechanical ventilation systems, and others) [5, 6]. Other mechanisms of transmission are aerial dispersion of opportunistic or environmental microorganisms and endogenous dissemination of commensal or opportunistic pathogens [7–9].

Although the role of the inanimate hospital environment in the spread of HAI has been controversial, nowadays molecular biology methodologies are being used to identify pathogens, measure the quality of environmental and hand hygiene over time, and establish a link between outbreaks and cross-transmission events, according to geographic and temporal variables [8].

Currently, changes in morbidity and mortality patterns due to aging of the world population, treatments with immunosuppressive drugs, and the use of invasive devices (particularly long-term ones) have led to a rise in the need of healthcare facilities for patients who are more susceptible to opportunistic infections [10]. Environmental disturbances associated with construction activities near health institutions pose additional airborne and waterborne disease threats for those patients who are at risk for healthcare-associated fungal infections [2]. Particularly, hospitalized patients could be exposed to infective fungal propagules such as microconidia and small hyphal fragments of* Histoplasma capsulatum* that thrive in bat and bird droppings, deposited in the surrounding hospital recreational areas.

Thus, the aims of this paper were to review the reported cases of* H. capsulatum* infections in healthcare settings, in order to propose the different factors that could be related to healthcare-associated histoplasmosis and discuss the features that could favor the presence of this fungus in the hospital environment.

## 2. Etiologic Agents of HAI

The etiologic agents of HAI are mainly bacteria (85%) and fungi (13%), in contrast to viruses and parasites that are rarely reported. Some environmental factors have been identified to increase the risk for fungal HAI, particularly the use of medical devices, like central venous and urinary catheters; the presence of severe cuts, wounds, and burns; stays in the intensive care unit, surgery, and hospital reconstruction works [4].

Host factors, such as extremes of age and underlying diseases, human immunodeficiency virus/acquired immune deficiency syndrome (HIV/AIDS), malignancy, and transplants, can increase susceptibility to infection, as well as a variety of medications that alter the normal flora, like antimicrobial agents, gastric acid suppressants, steroids, antirejection drugs, antineoplastic agents, and immunosuppressive drugs [2].

Most HAI associated with fungi are caused by* Candida* spp. These infections usually come from an endogenous source. However, cross-transmission via the hands of healthcare workers or contaminated devices can occur [11]. HAI outbreaks by other yeasts, such as* Malassezia* spp.,* Saccharomyces* spp., and* Trichosporon* spp., have also been identified in newborns, patients with hematologic malignancies, and transplant recipients [12–18]. Mechanical ventilation, duration of hospital stay, prolonged use of intravascular catheters, parenteral lipid formulations, and prior exposure to broad spectrum antibiotics (including antifungal therapy) are important predisposing conditions identified in these outbreaks [13, 14, 16–18].

The occurrence of invasive fungal infections (IFIs) depends on several factors like the time of exposure to an infectious agent, the patient's immune status, the pathogen's virulence factors, and the host-pathogen interaction [19]. IFI associated with healthcare is mainly caused by opportunistic fungi, from endogenous or environmental sources, which form biofilms in fomites and abiotic surfaces [9].

Species of filamentous fungi, such as* Aspergillus* spp. [20, 21],* Rhizopus* spp. [22],* Rhizomucor* spp. [22, 23],* Absidia corymbifera* [22, 24],* Fusarium* spp. [25–27],* Paecilomyces* spp. [28, 29],* Curvularia* spp. [30],* Phialemonium* spp. [31–34], and* Scedosporium* spp. [35–37], have been particularly associated with HAI in patients with hematologic diseases. The most common sources reported in the above-mentioned filamentous fungal infections were contamination of medical supplies, like intravenous solutions, contact lens solutions [38, 39], bandages [24], pressure cuffs, and invasive devices (endotracheal tubes) [11, 21–23, 40]. Besides, other species of fungi such as* Aureobasidium* spp. [41],* Trichosporon* spp. [42, 43],* Rhodotorula* spp. [44–46], and* Phaeoacremonium parasiticum* [47] have been implicated in nosocomial pseudooutbreaks through contamination of endoscopes.

A very important opportunistic fungus,* Pneumocystis jirovecii*, has also been associated with HAI by person-to-person airborne transmission [48–58]. Infection by* P. jirovecii* presents as an interstitial pneumonia in immunocompromised hosts, particularly HIV patients; in this group, pneumocystosis is considered an AIDS-definitory condition, when CD4+ T lymphocytes are below 200 cells/*μ*L [59]. Currently, an increase of pneumocystosis in non-HIV patients is being observed, especially in patients with transplants, individuals with autoimmune disorders or malignancies, and those using immunosuppressive treatments, like steroids and immunobiological drugs [50, 55, 60–62]. Molecular biology techniques have detected a high prevalence of colonization (10–55%) in immunocompromised patients and in the general population. Individuals colonized by* P. jirovecii* can be considered reservoirs and therefore contribute to the transmission of this pathogen among immunosuppressed patients in the hospital environment [62].

Other important respiratory pathogens, such as* Blastomyces dermatitidis*,* Paracoccidioides brasiliensis*, and* H. capsulatum*, have never been associated with infections in the hospital environment; however,* B. dermatitidis* has been found in pseudooutbreaks associated with contaminated bronchoscopes [63].

## 3. *H. capsulatum* Infection

*H. capsulatum* is a dimorphic fungus with a mycelial saprobe-geophilic morphotype (infective M-phase), usually found in bat and bird guano, and a yeast morphotype (parasitic and virulent Y-phase) preferentially located within phagocytes. Infection occurs through inhalation of aerosolized M-phase propagules, mainly microconidia and hyphal fragments, accumulated in confined spaces usually inhabited by bats or birds [64].

There are eight genetic populations of* H. capsulatum* distributed worldwide [65], between the latitudes 54° North [66] and 38° South [67], suggesting a broad geographic dispersion of the pathogen.* H. capsulatum* has been found in ecological niches with special conditions: air and soil temperatures (18–28°C), humidity (>60%), and darkness (fosters sporulation). Particularly, this fungus needs the presence of high concentrations of nitrogen and phosphorus for the M-phase growth, in addition to other micronutrients, which are plentiful in bat or bird guano [64, 68–70]. Besides, this fungus' ubiquitous distribution in nature (soil, treetops, yards, and public parks, among others) makes it feasible to find the M-phase in open spaces, either in rural or urban areas around hospitals [64, 70]. In a large outbreak that occurred in Acapulco, Mexico, the presence of the fungus was revealed in ornamental potted plants, containing organic material known as compost supplemented with bat guano that is used as fertilizer [70].

Histoplasmosis is a systemic mycosis preferentially distributed in endemic areas of the Americas. Most* H. capsulatum* infections are asymptomatic. A low number of individuals develop pneumonia, which is the main clinical form in immunocompetent patients (primary pulmonary histoplasmosis) with distinctive histopathological features, like chronic granulomatous infiltrate [69]. Epidemic outbreaks of histoplasmosis are related to occupational exposure or recreational activities and affect individuals worldwide [66, 71–73]. However, this disease is one of the most common opportunistic infections among HIV/AIDS patients with CD4+ T lymphocytes below 150 cells/*μ*L (known as AIDS-definitory condition), who may develop severe and fatal disseminated histoplasmosis [59]; approximately 30% of these patients die from this infection [74–76].* H. capsulatum* infections have also been described in patients with transplants [77, 78], invasive devices, and/or surgical implants [79–81].

*H. capsulatum* shares some features with the etiologic agents of HAI, bacteria or fungi, which support the nosocomial involvement of* H. capsulatum* infection: worldwide distribution (facilitated by flying reservoirs), its ubiquity, production of aerosolized infective propagules that spread the fungus in the environment and favor the infection by the respiratory pathway, development of biofilm and quorum-sensing (QS) events, and opportunistic behavior in immunosuppressed hosts.

## 4. Biofilm Formation

It is estimated that 95% of the microorganisms found in nature are attached in biofilms [82]. Over 60–65% human infections involve the formation of biofilms by normal commensal flora or nosocomial pathogens [83–89]. A biofilm is a complex structured community of microorganisms, surrounded by an extracellular matrix of polysaccharides, adhering to each other over a surface or interface [82]; sometimes protein-like adhesins of the pathogen are also involved in biofilm formation [90]. Biofilms constitute a potential source of chronic, recurrent infections and cross-contamination events [7, 89]. Microorganisms in biofilms are protected from the host's immune system and may be 1,000-fold more resistant to antibiotics than planktonic cells [91], due to poor penetration of drugs, low growth rate, and development of the microorganism's resistant phenotypes within biofilms [84, 92].

Fungal biofilms have been found not only in wild soil and water, but also in urban environments, like piping systems, water reservoirs, and constructions, and in healthcare equipments [8, 85, 93–96]. Among the fungal biofilms found on these surfaces, the medically important fungi, such as* Candida* spp.,* Aspergillus* spp.,* Cryptococcus* spp.,* Rhodotorula* spp.,* Penicillium* spp.,* Sporothrix* spp.,* Acremonium* spp., and* Paecilomyces* spp., must be highlighted [8, 90, 93–98].

In medical devices,* Candida* spp. is the most common fungi associated with biofilm formation, usually with endovascular and urinary catheter-related infections in intensive care units, resulting in invasive candidiasis with high mortality [99–101]. The distribution of* Candida* species is variable and in recent years non-*albicans Candida* species have been frequently found in patients with hemodialysis catheter-related candidemia [102].

The presence of biofilms has also been described in ventriculoperitoneal (VP) shunts in patients with* Candida* spp.,* Cryptococcus neoformans,* and* Coccidioides immitis* meningoencephalitis. These biofilms were associated with recurrent peritonitis and meningitis [88, 103]. Various fungi have been able to form biofilms on abiotic surfaces in experimental models, such as* A. fumigatus* [104],* M. pachydermatis* [105],* Blastoschizomyces capitatus* [106],* Candida* spp. [107],* Pneumocystis* spp. [108],* Rhodotorula* spp. [109, 110],* C. neoformans* [111],* S. cerevisiae* [112],* Fusarium* spp. [113],* T. asahii* [114], and zygomycetes [115].

Epidemiological surveillance definitions of HAI include surgical site infections associated with surgical implants or medical indwelling devices, when they occur within 30–90 days after the surgical procedure [4]. Clinically,* H. capsulatum* infections have been identified in individuals with invasive devices or surgical implants, and some authors have described endovascular histoplasmosis in patients with vascular prosthetic or synthetic implants [80, 81, 116–119]. Usually, the diagnosis is made by isolation of the fungus in vegetation or over synthetic materials. In addition, histopathological observation has revealed fibrin, large aggregates of yeast cells, mild chronic inflammatory cell infiltrates (predominantly macrophages) [80, 116, 118], and* H. capsulatum* hyphae (M-phase) in a few cases [117]. Furthermore,* H. capsulatum* endocarditis has also been described in native heart valves [118–120]. The aforementioned factors suggest the ability of* H. capsulatum* Y-phase to form biofilms in vivo (human solid organs and medical devices). Recently, it was described that* H. capsulatum* is able to form biofilms on abiotic surfaces [121]. Besides,* H. capsulatum* yeasts have been found clustered in the cells of bats' spleen, lung, and liver and in the lamina propria of intestine villi [122].

There are some reports of* H. capsulatum* peritonitis associated with infected catheters in patients with end-stage renal disease under continuous ambulatory peritoneal dialysis [123–128]. All of these peritoneal histoplasmosis' cases occurred in residents from an endemic area, in a period longer than 90 days, in contrast with the epidemiological definition of HAI. Thus, continuous exposure to the fungus' infective M-phase propagules appears to be an important risk factor, since no other epidemiological feature could be associated with these cases.

Veeravagu et al. [129] reported a case of* H. capsulatum* meningitis associated with a VP shunt that was diagnosed two days after surgery. It is noteworthy that the patient did not come from an endemic area. Furthermore,* H. capsulatum* was isolated from the VP shunt tip and the surgical instruments, so this could be considered a nosocomial histoplasmosis.

Currently, it is unknown if* H. capsulatum* is able to form biofilms in its filamentous form, which could contaminate hospital environments, medical devices, and supplies, facilitating the direct inoculation of the infective form through cross-contamination. However, it is not a farfetched idea, because biofilms have been described in filamentous fungi, such as* Aspergillus* spp. [104] and zygomycetes [115].

## 5. Quorum Sensing (QS)

QS is a mechanism of microbial communication dependent on cell density that can regulate several behaviors in bacteria, such as secretion of virulence factors, biofilm formation, survival, and bioluminescence. Fungal QS systems were first described in the pathogenic fungus* C. albicans*, with important signaling molecules, called farnesol and tyrosol (alcohols derived from aromatic amino acids), which control fungal growth, morphogenesis, and biofilm formation, inducing detrimental effects on host cells and other microbes. The concentration of these alcohols increases proportionally to the microbial population and, after reaching a critical threshold, a regulatory response is triggered leading to the coordinated expression or repression of QS-dependent target genes in the entire microbial population [130].

QS activities have also been described in other fungi, such as* H. capsulatum* [131],* Ceratocystis ulmi* [132], and* Neurospora crassa* [133]; however, the molecules responsible for such activities have not yet been purified. In* H. capsulatum*, regulation of *α*-(1,3)-glucan synthesis in the Y-phase cell wall has been shown to occur in response to cell density [134].

Albuquerque and Casadevall [130] proposed that fungal QS molecules are not only a product of fungal catabolism, but they should have some characteristics: to accumulate in the extracellular environment during fungal growth at a concentration proportional to the population cell density restricted to a specific stage of growth, to induce a coordinated response in the entire population once a threshold concentration is reached, and to reproduce the QS phenotype when added exogenously to the fungal culture. More research about these molecules is needed to elucidate the QS mechanisms in each fungus model, involving different pathogenic events, including biofilm formation.

## 6. *H. capsulatum* Infection in Drug-Induced Immunocompromised Individuals

IFIs related to immunosuppression caused by drugs in patients with transplant occur because cellular immunity is modified, usually within the first six months posttransplant. During this period, the IFI acquired an opportunistic nature and emerged as HAI [135–137]. After six months posttransplant, patients usually remain stable and continue receiving immunosuppressive drugs at low doses. Thus, they are susceptible to common infections acquired in the community [79, 135–137].

*H. capsulatum* infections have been identified in solid organ transplant (SOT) recipients [138, 139]. However, a low frequency of histoplasmosis related to HAI has been observed in the first six months posttransplant. Freifeld et al. [138] identified nine cases of pulmonary histoplasmosis in SOT recipients in a period of 30 months, but only four patients developed the disease in the first six months posttransplant. In a 10-year cohort study, Cuellar-Rodriguez et al. [140] found only three cases of histoplasmosis in SOT recipients in the first six months posttransplant; however, eleven cases were identified after the first year posttransplant.

Other authors evaluated the incidence of IFIs in SOT recipients, in a 5-year cohort study [141, 142]. Of the 1,208 cases of IFI, histoplasmosis was diagnosed in 48 patients, where 18 cases (37.5%) occurred in the first six months posttransplant, particularly in kidney, liver, and kidney-pancreas transplants [141]. In general, these patients were receiving immunosuppressive drugs, like tacrolimus, sirolimus, mycophenolate mofetil, and steroids [138, 140, 142].

A more recent study about IFIs identified an increase in the number of histoplasmosis cases in SOT recipients [142]. Among the 70 cases of IFIs reported, 52 (80%) were diagnosed as histoplasmosis, in a 5-year period. The median time from transplant to the diagnosis of this fungal disease was one year. Five SOT recipients developed histoplasmosis within 30 days of transplant; two patients acquired the infection from their donated organs, and three patients developed pulmonary histoplasmosis irrespectively of the transplanted organs [142]. In rare cases, histoplasmosis has also been diagnosed in patients treated with immunobiological molecules, like different monoclonal antibodies [143, 144].

## 7. Fungal Respiratory Infections in the Hospital Environment

Inadvertent exposure to opportunistic environmental and airborne pathogens can result in infections with significant morbidity and mortality [9]. Fungal infections can range from mild to life-threatening; they vary among mild skin rashes, fungal pneumonia, meningitis, and IFIs. In the hospital, the most common fungal HAI are caused by* Candida* spp. and* Aspergillus* spp. [19].

Airborne infections in susceptible hosts may result from exposure to environmental microorganisms that are ubiquitous in nature, growing in soil, water, dust, or organic matter [2, 9]. Spores or hyphal fragments of fungi usually lie scattered in the environment, especially near decomposing organic matter.* Aspergillus fumigatus* is the species most often associated with pulmonary IFIs [3]. Infection occurs after inhalation of conidia stirred up from construction or renovation works in the hospital. The main risk factor for this HAI is the concentration of* Aspergillus* conidia in the air [2, 145–149], and the most susceptible individuals are hematopoietic stem cell transplant recipients, neutropenic patients, and those with hematologic malignancies [136, 145, 150–153].

Infections due to* C. neoformans*,* H. capsulatum*, or* C. immitis* can occur in healthcare settings if the nearby ground is disturbed and a malfunction of the facility's air-intake components allows these pathogens to enter the hospital ventilation system [9]. Several outbreaks of histoplasmosis have been associated with disruption of the environment [67, 72, 154].* H. capsulatum* contaminated environments related to bat and bird colonies living in abandoned buildings and on treetops could disperse the fungus around the hospital. Under this statement, the dispersion of* H. capsulatum* infective propagules could represent a potential risk factor for hospital-acquired histoplasmosis, especially in individuals hospitalized in units lacking adequate air quality control.* H. capsulatum* has never been identified in air quality studies from hospital settings [152]. This could be explained by the difficulties in this fungus' isolation, including prolonged culture growth in laboratory conditions, special nutritional needs, and culture inhibition by the presence of other fast-growing fungi [64, 68].

## 8. Molecular Biology as a Diagnostic Tool in HAI

Hospital-acquired pneumonia represents one of the most difficult treatment challenges in infectious diseases. Many studies suggest that the timely administration of appropriate pathogen-directed therapy could be lifesaving. However, results of bacterial cultures and antimicrobial susceptibility testing can take 48 hours or longer, but some fungi may not even be able to grow in the first week after culturing.

Nowadays, physicians rely on clinical and epidemiological factors to choose an initial empiric therapy for HAI. A number of rapid molecular tests have been developed to identify pathogens and the bases for most molecular assays are polymerase chain reaction and nucleic-acid-sequence-based amplification. These methodologies offer the promise of dramatically improving the ability to identify pathogens in respiratory tract specimens with high sensitivity and specificity. Data from such applications can also be electronically integrated into shared molecular databases, where clinicians and epidemiologists could ascertain local, regional, national, and international trends [155].

Molecular identification of fungi in hospitals has been scarcely described [156–158]. Lo Passo et al. [156] reported transmission of* Trichosporon asahii* by an endoscopic procedure, when isolated from an esophageal ulcer.* T. asahii* isolates were genotyped by restriction fragment length polymorphism and random amplification of polymorphic DNA, confirming the endoscopic device as the source of transmission.

Notwithstanding, there are some undefined issues regarding the use of these molecular biology tools.

- Molecular assays have been used mainly for bacteria and viruses, leaving aside the importance of other microorganisms, such as pathogenic fungi; however, there are specific markers for almost every fungal pathogen, which have recently improved the molecular diagnostic bundle for HAI [157].
- The significance of finding a pathogen's DNA in respiratory tract specimens, in the absence of a positive culture, will show different airway ecology from what it is known, and it exposes the inability to distinguish between infecting and colonizing organisms [155].
- The complexities of the pulmonary microbiome and its metagenomic diversity represent a great challenge with many unanswered questions remaining [155].
- New procedures combining molecular biology techniques and environmental sampling of air have revealed some fungal pathogens living in the hospital surroundings [158], which may be relevant for the acquisition of respiratory HAI.

## 9. Conclusions

*H. capsulatum* infection associated with healthcare has been linked to medical devices and surgical implants. The M-phase of* H. capsulatum* may be present in hospital settings, as this fungus adapts to different types of climates and has great dispersion ability. Although conventional pathogen identification techniques have never identified* H. capsulatum* in the hospital environment, histoplasmosis HAI cases have been reported in the last decades. Molecular biology procedures could be useful in this fungus' identification in the air of hospitals and in the diagnosis of this mycosis. More research is needed about* H. capsulatum* involvement in HAI, since it causes a severe and often fatal disease in immunocompromised individuals.
